# Supplementary material for: Structure, dynamics and roX2-lncRNA binding of tandem double-stranded RNA binding domains dsRBD1,2 of Drosophila helicase Maleless
Source: Nucleic Acids Res. 2019 Feb 26;47(8):4319–33. doi: 10.1093/nar/gkz125 (PMC6486548; doi:10.1093/nar/gkz125)
Supplement: Supplementary Data [file gkz125_supplemental_file.pdf]

Structure, dynamics and roX2-lncRNA binding of tandem double-stranded RNA binding domains dsRBD1,2 of *Drosophila* helicase Maleless

Supplementary material

Supplementary Tables

rox2-SL3.fw	TTACATATAGCTTTAGAGATCGTTTCG
rox2-SL3.rv	GCTTGATTTTGCTTCGGAGA
rox2-SL7.fw	GACGTGTAAAATGTTGCAAATTAAG
rox2-SL7.rv	TGACTGGTTAAGGCGCGTA
7SK.fw	GATAACCCGTCGTCATCCAG
7SK.rv	AGTAATTCTGCCTGGCGTTG

Table S1. Different primers for qPCR used in this study.

Sample	dsRBD1,2	SL7^{18mer}	dsRBD1,2+ SL7^{18mer} complex
**a. Sample Details**			
Organism	*E. Coli* BL21 (DE3)	synthetic	-
Source	*this work*	IBA	*this work*
Description	P24785(1-257) with additional GA at the N-terminal after TEV cleavage	doubled-stranded RNA: AGACGUGUAAAAUGUUGC GUAACGUUUUACGCGCCU	1:1 mixture of RNA and protein
Molecular masses from chemical composition (Da)	28.42	11.94	-
Loading concentration (mg ml^{-1})	5.0	1.0	1.0 (1:1 molar-ratio)
Injection volume (µl)	40	40	40
Solvent composition	200 mM NaCl, 20 mM $NaPO_4$, 1mM DTT, pH 6		
**b. SAS data collection parameters**			
Source and instrument	Grenoble ESRF BM29 with Dectris Pilatus 1M		
Wavelength (Å)	0.9919		
Sample-detector distance (m)	2.867		
q-measurement range (nm^{-1})	0.0348–4.9417	0.0348–4.9417	0.0348–4.9417
Radiation damage monitoring	frame-by-frame comparison		
Exposure time (s) & number	1.0 ×10		
Sample temperature (ºC)	20		
**c. Software employed for SAS data reduction, analysis and interpretation**			
SAXS data processing	PRIMUS from ATSAS 2.8		
Molecular graphics	VMD		
**d. Structural parameters**			
**Guinier analysis**			
I(0) (raw)	66.26±0.13	26.38±0.13	40.73±0.09
R_g (nm)	3.23±0.13	1.83±0.51	3.11±0.31
q-range (nm^{-1})	0.058–0.313	0.399–0.709	0.096–0.374
Coefficient of correl. R^2	0.99	0.76	0.92
**P(r) Analysis**			
I(0) (cm^{-1})	66.44±0.15	27.73±0.36	41.25±0.15
R_g (nm)	3.34±0.02	2.10±0.05	3.34±0.03
d_{max} (nm)	12.9	8.5	13.3
q-range (nm^{-1})	0.058–2.004	0.398–4.369	0.096–2.0004
GNOM total est.	0.447	0.468	0.643
**f. Atomistic modelling**			
Method	EOM	-	-
q-range for fitting (nm^{-1})	0.0–5.0	-	-
Domains configurations	dsRBD1:(1–82), dsRBD2:(158-259)	-	-
Flexible linker definition	83–157	-	-
Number of starting models	10,000	-	-
χ^2	5.455	-	-
Constant subtraction	Yes	-	-
Ensemble avg. R_g	3.43	-	-
Ensemble avg. C_α end-to-end distance	6.27	-	-
Ensemble avg. d_{max}	10.61	-	-
**g. Data and model deposition IDs**			
SASBDB	SASDF52	SASDF72	SASDF62

Table S2. SAXS data collection and processing statistics.

Sample	N (sites)	K_D (μM)	ΔH (kJ/mol)	ΔG (kJ/mol)	-TΔS (kJ/mol)
$SL7^{18mer}$ vs dsRBD2	0.70 ± 0.01	2.71 ± 0.19	-86.0 ± 1.5	-31.30	54.7
$SL7^{18mer}$ vs dsRBD1,2	0.84 ± 0.03	3.18 ± 0.48	-145.0 ± 8.2	-30.90	114.5
$SL7^{14merLoop}$ vs dsRBD2	0.93 ± 0.05	5.92 ± 1.31	-87.7 ± 8.1	-29.51	58.76
$SL7^{14merLoop}$ vs dsRBD1,2	0.95 ± 0.03	4.66 ± 0.71	-100.0 ± 7.2	-30.00	70.20

Table S3. Isothermal titration calorimetry data for RNA binding to MLE dsRBD1,2. Errors calculated from error propagation of fitting errors of two experiments.

Supplementary figures

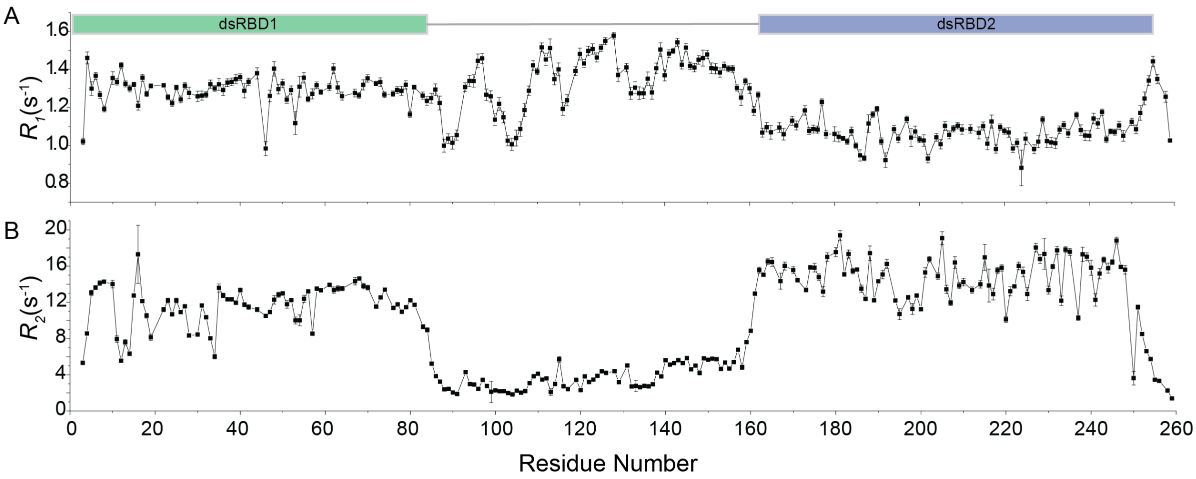

Figure S1: (A) & (B) ^{15}N transverse relaxation analysis of dsRBD1,2 in the free form suggesting that the two domains tumble independently in solution.

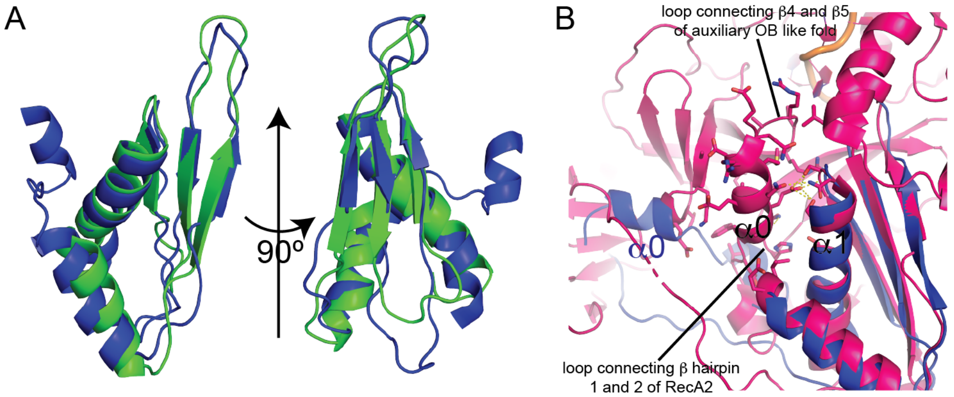

Figure S2: (A) Comparison of dsRBD1 (green) and dsRBD2 (blue) NMR structures. The two domains superpose well with an RMSD of 1.2 Å. dsRBD2 contains an extra α0 helix in the

structure. (B) Superposition of dsRBD2 structures as determined by NMR (blue) and crystallography (magenta) (in the MLE_{core} domain, PDB ID: 5AOR) showing the packaging of α0 helix in the crystal structure. The α0 helix in the NMR structure is flexible.

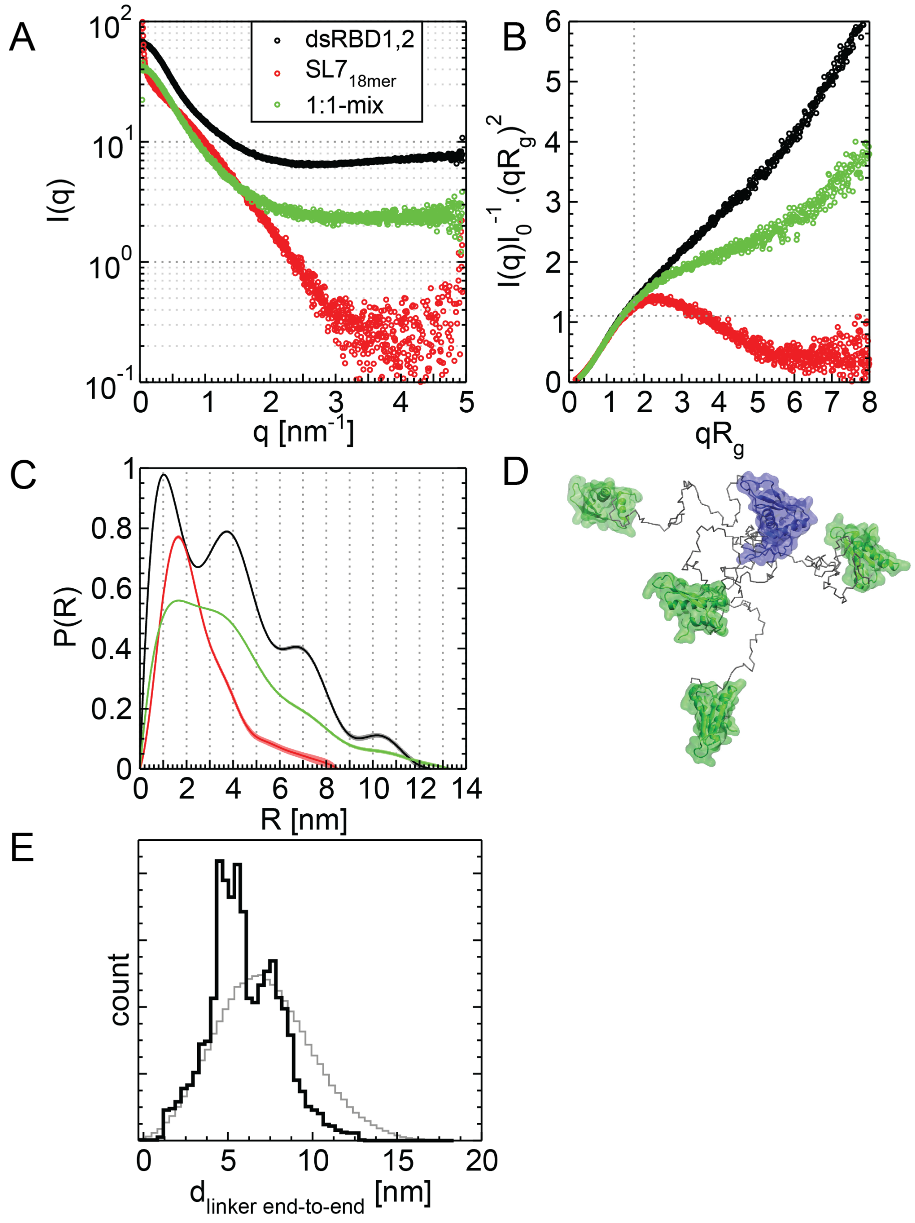

Figure S3: (A) SAXS data for dsRBD1,2 (black), $SL7^{18mer}$ (red) and 1:1 complex of dsRBD1,2 and $SL7^{18mer}$ (green). (B) Corresponding Kratky plots and (C) pairwise distribution functions for the three scattering curves. Note that the sinusoid features of the apo P(R) curve above ~5 nm is an artefact of modelling. (D) 4 representative structures of dsRBD1,2 produced from EOM analysis. (E) Distribution of end-to-end linker distances (black) versus the initial input random-coil distribution (grey) from EOM analysis.

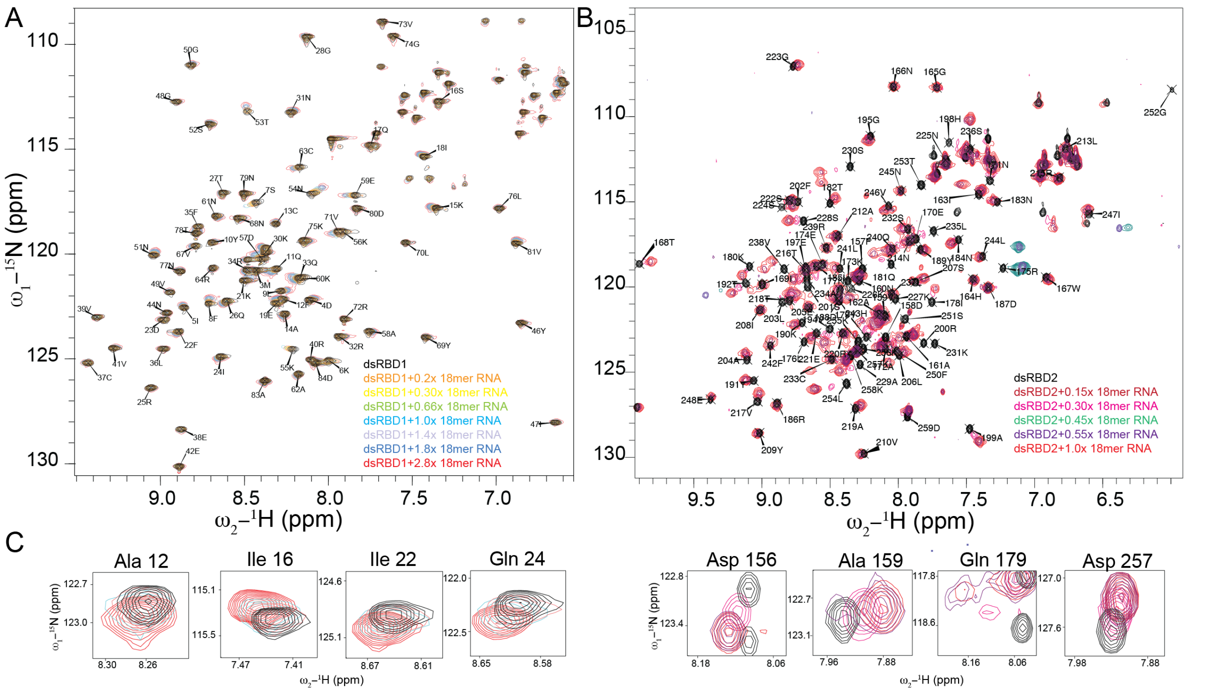

Figure S4: (A, B) ^{1}H, ^{15}N HSQC NMR titration of individual dsRBD domains with SL7^{18mer} dsRNA. (C) Zoomed-up views of peaks showing shifts upon RNA titration.

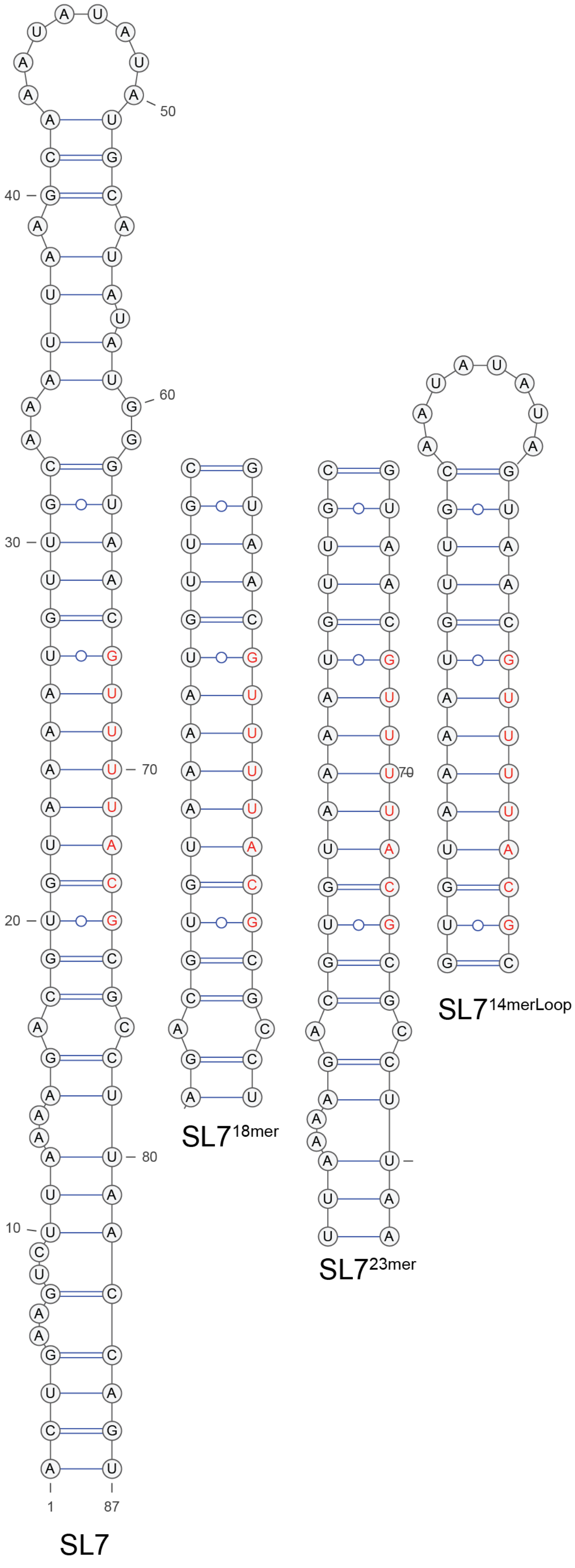
SL7
SL7 18mer
SL7 23mer
SL7 14merLoop

Figure S5: Different RNA's used in this study and derived from roX2 SL7 stem are shown. The roX-box region is indicated using red fonts.

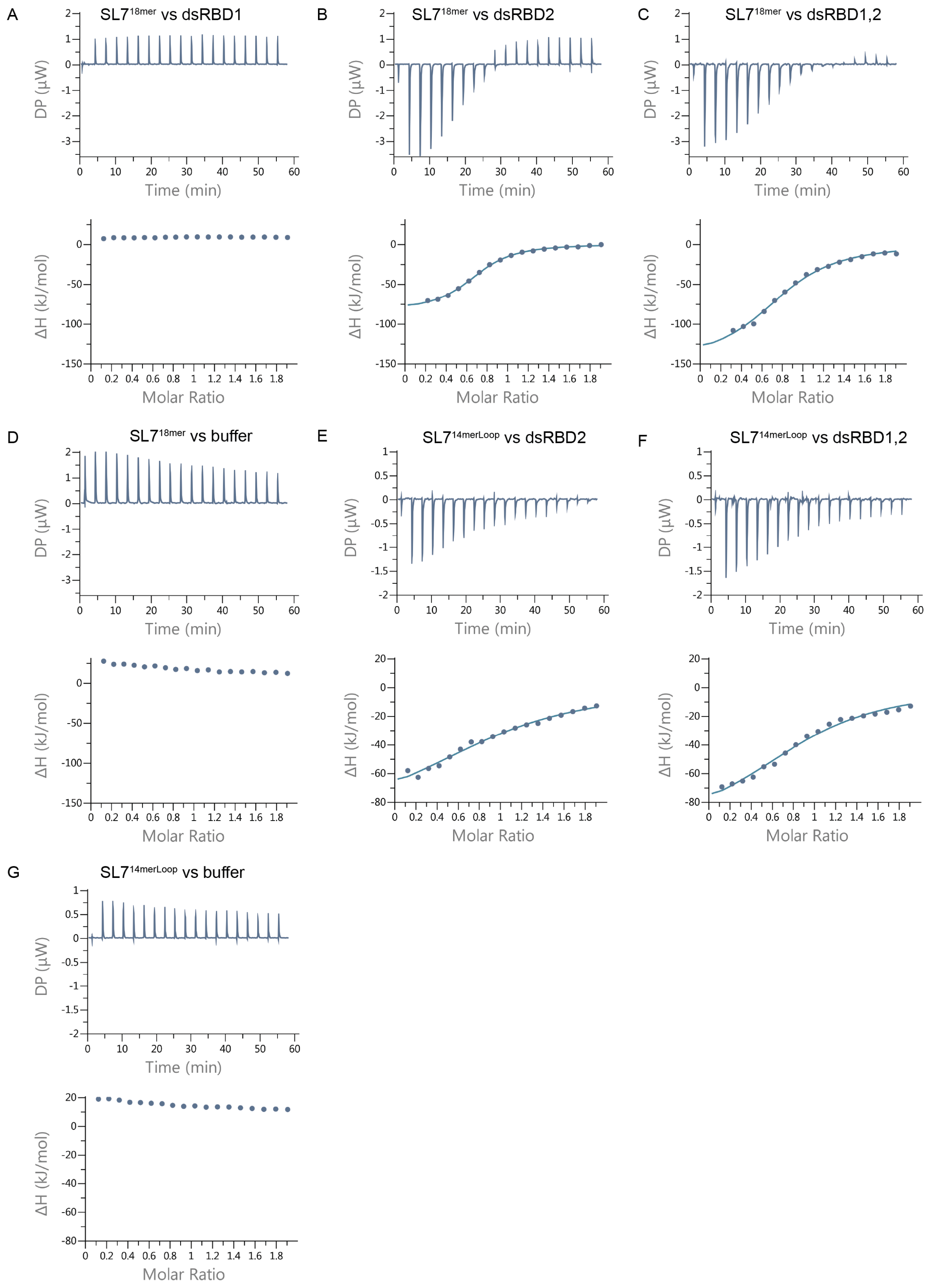

Figure S6: (A-G) Representative ITC curves for titration of SL7^{18mer} and SL7^{14merLoop} in dsRBD1, dsRBD2 and dsRBD1,2.

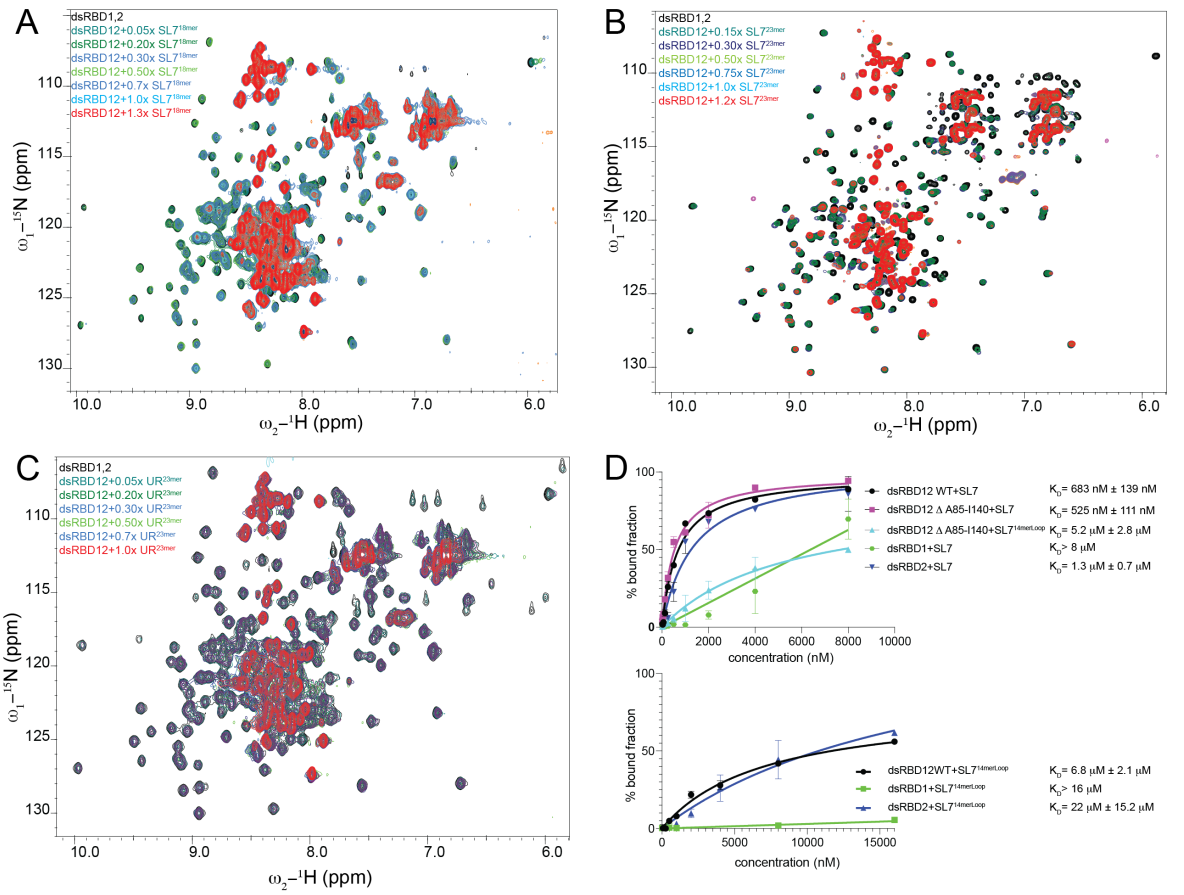

Figure S7: Full ^{1}H, ^{15}N HSQC NMR titration of dsRBD1,2 with (A) SL7^{18mer}, (B) SL7^{23mer} and (C) UR^{23mer}. All titrations show severe line broadening with increasing concentration of RNA except in the linker region. (D) Filter binding experiments of dsRBD1,2, dsRBD1, dsRBD2 and dsRBD1,2 (Δ A85-I140) with SL7 and SL7^{14merLoop} dsRNA. Error bars represent standard deviation of two replicates.

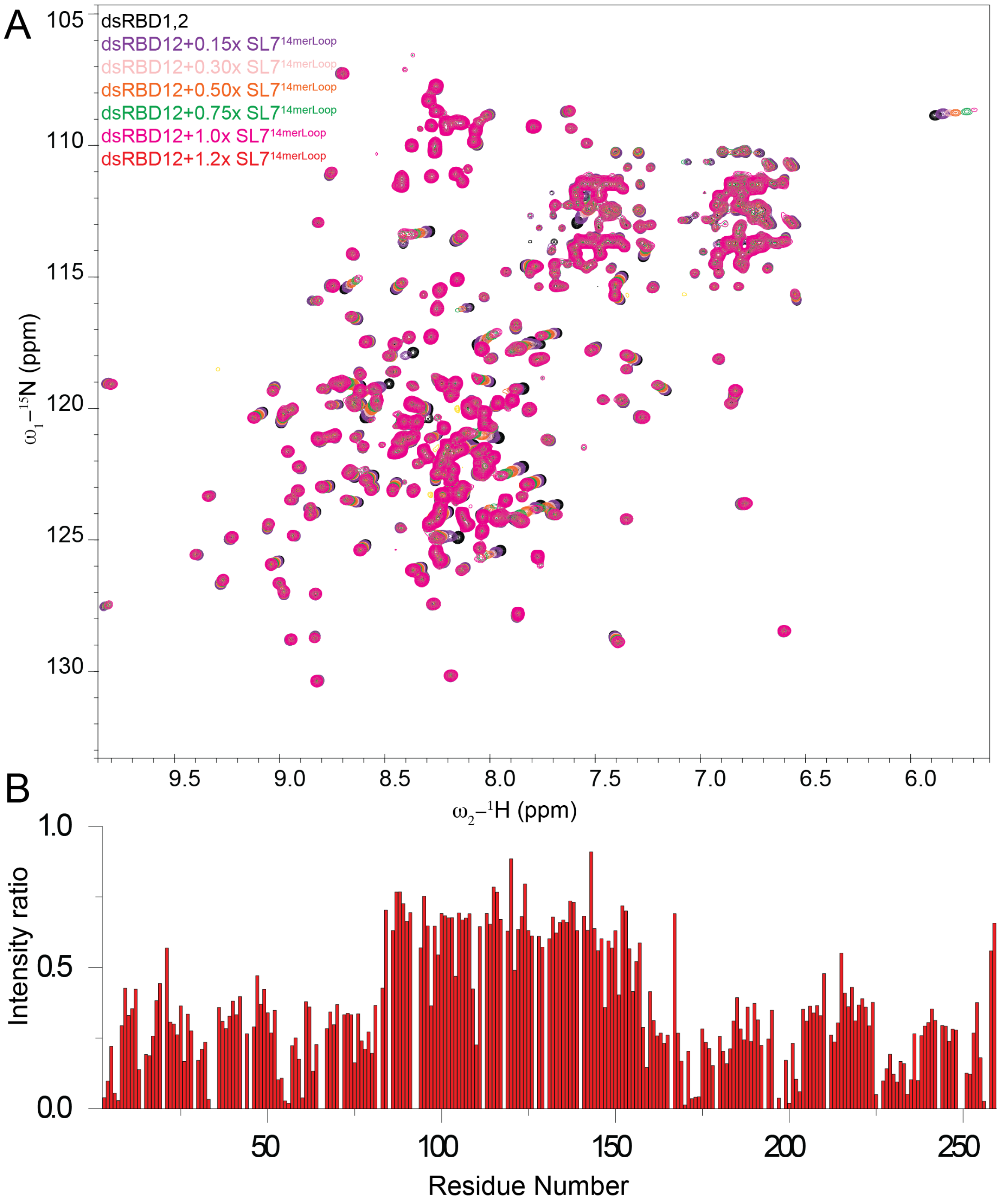

Figure S8: (A) ^{1}H, ^{15}N HSQC NMR titration of dsRBD1,2 with SL^{14merLoop}. (B) Intensity ratios of dsRBD1,2 + SL^{14merLoop} and dsRBD1,2 free showing a minor drop of intensity within the linker region and signifying its flexibility.

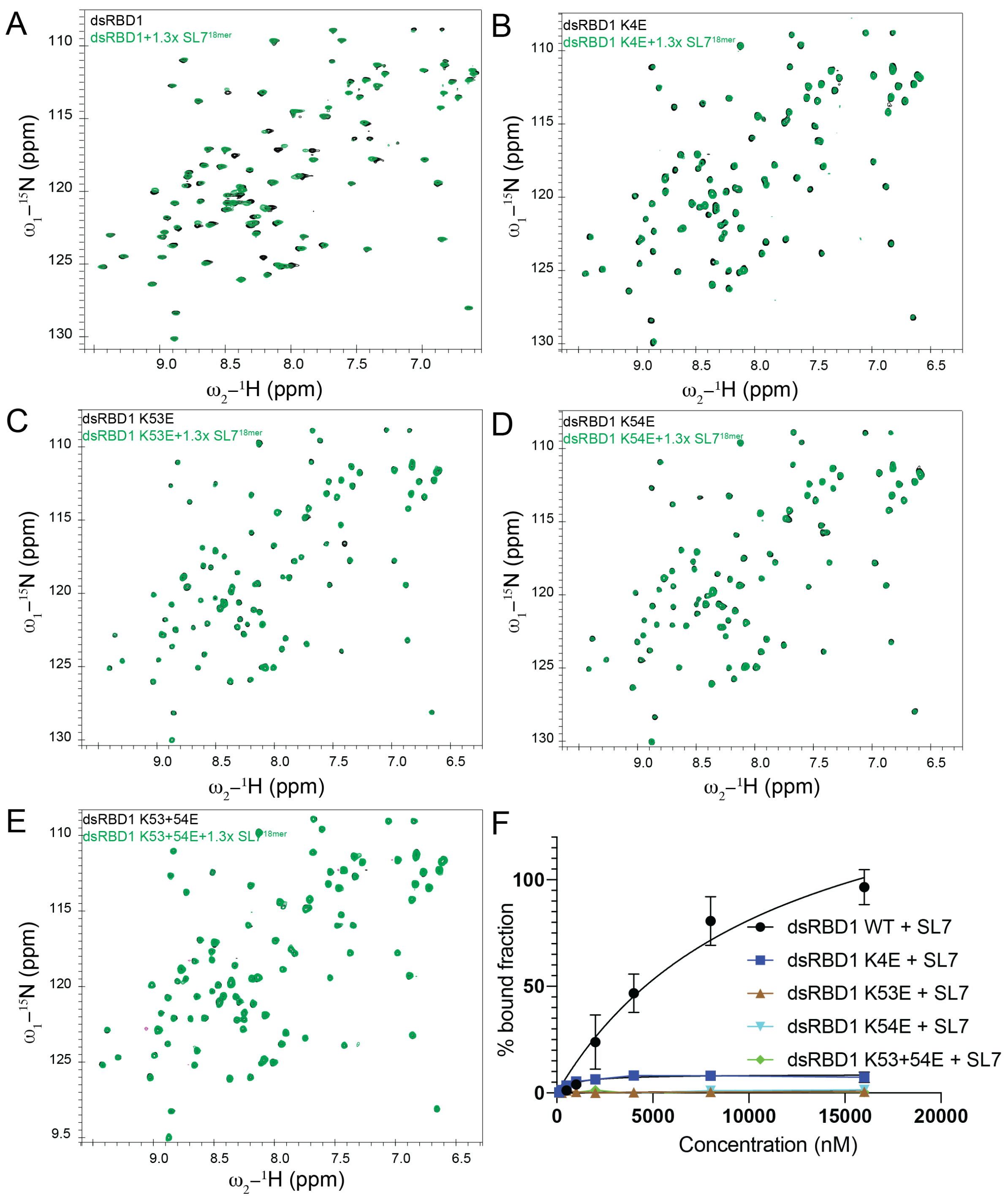

Figure S9: RNA binding of dsRBD1 mutants (A) dsRBD1, (B) dsRBD1 K4E, (C) dsRBD1 K53E, (D) dsRBD1 K54E and (E) dsRBD1 K53+54E upon titration with 1.3x $SL7^{18mer}$ RNA. (F) Filter binding experiments of individual dsRBD1 RNA binding mutants for SL7 binding. Only dsRBD1 WT shows binding with a K_D of 11.5 µM ± 8.1 µM.

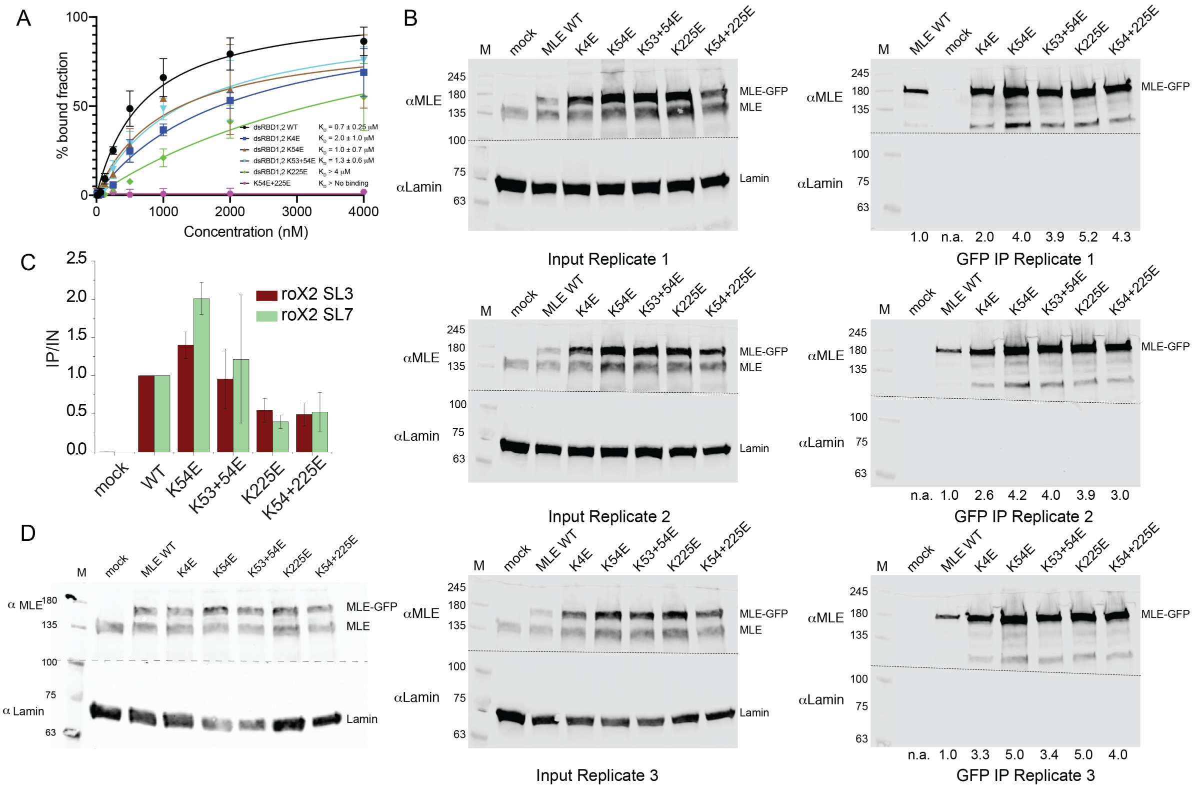

Figure S10: (A) Effect of dsRBD1 RNA binding mutations in the dsRBD1,2 context as determined by filter binding experiments. SL7 RNA was used for these experiments. The error bars represent standard deviation of two replicates (B) Western blot analysis of S2 cell lines stably expressing MLE-GFP and its dsRBD1,2 variants, which were used for three independent replicates of *in vivo* RNA immunoprecipitation experiments shown in (C). MLE-GFP levels in input (left) and GFP-immunoprecipitated fractions (right) were detected using anti-MLE antibody. Lamin served as loading control. The fraction of each immunoprecipitated MLE-GFP variant relative to MLE-GFP wild type is given. (C) *In vivo* RIP of MLE-GFP wild type or mutated in dsRBD1,2. Enrichment of roX2 by the MLE-GFP derivatives is shown relative to MLE-GFP wild type. Error bars represent average standard deviations for three independent biological replicates. (D) Western blot analysis of S2 cell lines stably expressing MLE-GFP and its dsRBD1,2 variants used for immunostaining studies. Anti-MLE antibody was used to detect endogenous MLE and MLE-GFP variants, respectively. Lamin served as loading control.
